# Effects of a Combined Chinese Herbal Medicine on Growth Performance, Intestinal Barrier Function, Immune Response, and Cecal Microflora in Broilers Infected with *Salmonella enteritidis*

**DOI:** 10.3390/ani14182670

**Published:** 2024-09-13

**Authors:** Changzhi Zou, Xin Xing, Shunxi Li, Xuelong Zheng, Jinshan Zhao, Huawei Liu

**Affiliations:** 1 College of Animal Science and Technology, Qingdao Agricultural University, Qingdao 266109, China; 20222103009@stu.qau.edu.cn (C.Z.); xingxin@stu.qau.edu.cn (X.X.); zhaojinshan@qau.edu.cn (J.Z.); 2 Guangrao County Livestock Development Service Center, Dongying 257000, China; 19854657565@139.com; 3 Pingdu Yunshan Animal Health and Product Quality Supervision Station, Qingdao 266700, China; lzjh88@126.com

**Keywords:** compound Chinese herbal medicine, broiler, *Salmonella enteritidis*, immune function, intestinal barrier

## Abstract

**Simple Summary:**

Bacterial enteritis caused by *Salmonella enteritidis* infection can reduce the growth performance and increase the mortality of broilers. Antibiotics were the mainstay of treatment for intestinal injury caused by *Salmonella enteritidis*, but the overuse of antibiotics has resulted in severe drug residues and the emergence of antibiotic-resistant bacteria. Chinese herbs contain natural compounds with high safety and medicinal value, making them good substitutes for antibiotics. Compared with use of a single Chinese herb, the rational combination of multiple herbs could produce synergistic effects to enhance therapeutic outcomes. In this study, the specific compound Chinese herbal medicine (CCHM) consisted of *Pulsatilla*, *Coptis chinensis*, *Fraxini cortex*, *Cortex phellodendri*, *Folium isatidis*, *Raddix paeoniae alba*, *Schisandra chinensis*, and *Fructus mume* in a ratio of 15:6:6:6:6:6:6:5. Our results showed that CCHM improved the growth performance, intestinal barrier function, immune response, and cecal microflora of broilers infected with *Salmonella enteritidis*.

**Abstract:**

This study investigated the effects of CCHM in drinking water on broilers infected with *Salmonella enteritidis*. One-day-old male Cobb 500 broilers (n = 300) were randomly assigned to five groups: a control (NC) group, a *Salmonella enteritidis* challenge (SE) group, an antibiotic (AB) group, a low dose of CCHM (CL) group, and a high dose of CCHM (CH) group. Each group had six replicate cages with ten broilers per cage. The broilers in the NC and SE groups were given normal drinking water. From days 12 to 18, the AB group received water treated with ciprofloxacin lactate injection (1 mL/L), while the CL and CH groups received water containing CCHM at doses of 5 mL/L and 10 mL/L, respectively. Broilers in all groups except the NC group were orally given *Salmonella enteritidis* daily from days 9 to 11. The experimental period was 28 days. The results showed that, compared with the SE group, the CL and CH groups showed improved growth performance; increased immune organ indices, expressions of ileal occludin and ZO-1 proteins, jejunal and ileal villus heights (except at day 19), and cecal *Lactobacillus* counts on days 19 and 28 (*p* < 0.05); and decreased jejunal and ileal lesion scores, ileal interleukin 1β (IL-1β) (except at day 19), interferon-γ (IFN-γ), interleukin 6 (IL-6) (except at day 19), secretory immunoglobulin A (slgA) and tumor necrosis factor α (TNF-α) (except at day 19) levels, serum *D*-lactic acid and diamine oxidase (DAO) (except at day 19) contents, jejunal and ileal crypt depths (except at day 19), and cecal *Salmonella* and *Escherichia coli* counts on days 19 and 28 (*p* < 0.05). On day 28, except for the levels of ileal interleukin 10 (IL-10), TNF-α, slgA, and serum D-lactic acid content, there were no differences among the NC, AB, and CL groups (*p* > 0.05). In conclusion, drinking water supplemented with CCHM alleviated the intestinal damage caused by *Salmonella enteritidis* infection and improved growth performance and cecal microbiota in broilers. The optimal addition rate of CCHM was 5 mL/L.

## 1. Introduction

*Salmonella enteritidis* is one of the most common pathogens in broilers, typically leading to severe intestinal diseases [1]. *Salmonella enteritidis* infection alters the gut microbiota and damages the intestinal barrier, which results in decreased growth performance, diarrhea, and increased mortality in broilers [2,3]. Antibiotics are the primary agents for the prevention and treatment of *Salmonella enteritidis* infection [4]. However, extensive antibiotic use causes the emergence of resistant bacteria and drug residues [5]. Therefore, there is an urgent need for novel treatment approaches to combat these resistant strains and residues.

Traditional Chinese herbs are considered as substitutes for antibiotics due to their lower toxicity and fewer side effects, as well as their ability to reduce residues in the body and their lower risk of inducing drug resistance [6,7]. The bioactivities of herbs are attributed to their chemical constituents, including organic acid, polysaccharides, flavonoids, alkaloids, and so on [8,9]. Many studies have shown the anti-inflammatory, antioxidant, and antimicrobial properties of these compounds, which contribute to immune modulation, enhancement of intestinal barrier function, and maintenance of intestinal microbiota balance [10,11]. The specific compound Chinese herbal medicine (CCHM) in this study consisted of *Pulsatilla*, *Coptis chinensis*, *Fraxini cortex*, *Cortex phellodendri*, *Folium isatidis*, *Raddix paeoniae alba*, *Schisandra chinensis*, and *Fructus mume* in a ratio of 15:6:6:6:6:6:6:5. *Pulsatilla* is a primary constituent of CCHM, known for its antioxidant and immune-modulating properties [12]. A study showed that *Pulsatilla* modulated immune responses and altered the composition of intestinal microbiota in Hungarian geese [13]. Berberine, the major active constituent in *Coptis chinensis* and *Cortex phellodendri*, is an isoquinoline derivative alkaloid with the ability to modulate intestinal microbiota and improve the growth performance of broilers [14,15,16]. Previous studies have shown that esculin, an active constituent of *Fraxini cortex*, exhibits inhibitory effects against *Salmonella enteritidis* and *Salmonella typhimurium* in vitro [17]. *Radix paeoniae alba* was reported to improve the intestinal flora in broilers with enteritis due to the antibacterial properties of its main bioactive constituents, such as *Radix paeoniae alba* polysaccharide [18]. Citric acid in *Folium isatidis*, *Schisandra chinensis*, and *Fructus mume* has an antibacterial effect and has been used to reduce *Escherichia coli* counts and promote intestinal villus growth in broilers [19,20,21,22]. Compared with the use of single Chinese herbs, the intricate composition of traditional Chinese herbal formulations facilitates synergistic interactions among their various constituents, thereby enhancing therapeutic efficacy [23,24]. Our previous research found that CCHM has an inhibitory effect on *Salmonella enteritidis* and protects intestinal epithelial cells against *Salmonella enteritidis* in vitro (unpublished data). However, the impact of CCHM on the growth performance and intestinal health of broilers infected with *Salmonella enteritidis* required further study. The present study aimed to evaluate the effects of CCHM on the growth performance, intestinal barrier function, immune response, and cecal microflora of broilers infected with *Salmonella enteritidis*.

## 2. Materials and Methods

### 2.1. Animal Ethical Approval

All experimental procedures were approved by the Animal Care and Use Committee of Qingdao Agricultural University (DKY20231011).

### 2.2. Materials

The ciprofloxacin lactate injection was purchased from Luoyang Huizhong Animal Medicine Co., Ltd., Luoyang, China.

The *Salmonella enteritidis* serotype CVCC3379 was purchased from the China Institute of Veterinary Drug Control (Beijing, China). *Salmonella enteritidis* pre-culture was transferred to xylose lysine desoxycholate agar (XLD) medium (Solarbio Technology Co., Ltd., Qingdao, China) at 37 °C under aerobic conditions for 24 h, and colonies with optimal growth were selected for further purification.

A total of 100 g of herbs was used to prepare CCHM according to the ratio of 15:6:6:6:6:6:6:5, which was then boiled twice with water (3–4 times the weight of the herbs each time). Firstly, the mixture was brought to a vigorous boil and then kept at a gentle boil. The first decoction was boiled for 1 h and filtered through gauze. The second decoction was simmered on low heat for 40 min and filtered through gauze. The combined decoctions were further reduced until 100 mL of liquid medicine remained, achieving a concentration of 1 g/mL. After autoclaving, the solution was filtered through a 0.22-micron filter and stored at cold temperatures for later use.

### 2.3. Identification of the Chemical Constituents in CCHM

A volume of 100 µL of CCHM was extracted and thoroughly mixed with 400 µL of methanol using a vortex mixer for 10 min. Subsequently, the mixture was centrifuged at 8000× *g* for another 10 min to separate the supernatant. This clarified solution was then subjected to machine analysis. Sample solutions were analyzed using an UltiMate 3000 RS system coupled with a Q Exactive quadrupole Orbitrap mass spectrometer (Thermo Fisher Scientific Shier Technology Co., Ltd., Wuhan, China). High-resolution liquid chromatography data were initially processed using Compound Discoverer 3.3 (CD 3.3, Thermo Fisher Scientific), followed by database retrieval and comparison in mzCloud [25].

### 2.4. Experimental Procedure

A total of 300 one-day-old male Cobb 500 broilers, each weighing 44.16 ± 0.97 g, were used in a completely randomized design experiment. The broilers were divided into five groups: a normal control (NC) group, a *Salmonella enteritidis* challenge (SE) group, an antibiotic (AB) group, a low dose of CCHM (CL) group, and a high dose of CCHM (CH) group. Each group consisted of six replicate cages, with ten broilers per cage. The broilers in the NC and SE groups were given normal drinking water. From days 12 to 18, the AB group received water treated with ciprofloxacin lactate injection (1 mL/L), while the CL and CH groups were provided water containing CCHM at doses of 5 mL/L and 10 mL/L, respectively. The experimental period was 28 days. The *Salmonella enteritidis* used in the bacterial enteritis model was obtained following the method described by Zhen et al. [26]. From days 9 to 11, broilers in all groups except the NC group were orally gavaged with 1 mL of 1 × 10^9^ CFU *Salmonella enteritidis*; the NC group was orally gavaged with normal sterile medium in equivalent amounts (Table 1). The basal diet was formulated according to the Cobb Broiler Management Guide [27] (Table 2). All broilers had free access to feed and water. The dimensions of the cages used were 80 cm × 65 cm × 55 cm (length × width × height). The temperature in the chicken coop was 33 °C–35 °C for the first week and then gradually decreased by 2 °C each week until it reached 25 °C. The lighting program provided 23 h of light and 1 h of darkness during the experiment.

### 2.5. Growth Performance Measurement

Daily feed intake was measured on a replicate basis. After a 12 h fast, body weight was recorded on days 0, 9, 19, and 28 to calculate the average daily feed intake (ADFI), average daily gain (ADG) and feed:gain (F:G) ratio on a replicate basis. Daily mortality was observed and recorded.

### 2.6. Sample Collection

One broiler per cage was randomly selected for blood sampling and individual weighing on days 19 and 28. Blood samples were obtained from the jugular vein and transferred into centrifuge tubes. Serum was then obtained by centrifuging the samples at 3000× *g* for 10 min at 4 °C; afterward, samples were stored at −20 °C. Subsequently, selected broilers were euthanized using the cervical dislocation method. Post-euthanasia, the thymus was harvested from the ventral neck dissection, while the bursa of Fabricius and the spleen were collected following abdominal incision. All organs were then weighed. For histological analysis, ileum and jejunum sections were preserved in 4% buffered formaldehyde. Additionally, cecal contents and ileum mucosa were harvested for subsequent investigation.

### 2.7. Intestinal Lesion Scores

On days 19 and 28, lesion scores ranging from 0 to 6 were assigned to the jejunum and ileum using the Chiu/Park scoring method, where higher scores indicated greater degrees of damage [28].

### 2.8. Relative Immune Organ Weight

The weights of the bursa of Fabricius, thymus, and spleen were determined, and the immune organ indices were calculated using the following equation: immune organ weight (g)/body weight (kg) [29].

### 2.9. Analysis of Biochemical Indices

Serum concentrations of *D*-lactic acid and DAO were measured using enzyme-linked immunosorbent assay (ELISA) kits (Beijing FreeMore Bioscience Co., Ltd., Beijing, China). A 0.1 g sample of ileum mucosa was taken from ileum samples and homogenized with 0.9 mL normal saline. The concentrations of IL-1β, IL-6, IL-10, TNF-α, IFN-γ, and sIgA were then measured using ELISA kits (Beijing FreeMore Bioscience Co., Ltd., Beijing, China), and the protein concentration was measured using a BCA protein quantification kit (ACRO Biosystems Biotechnology Co., Ltd., Beijing, China), with results expressed per milligram of protein.

### 2.10. Western Blot Analysis of Protein Expression in Ileal Mucosa

Total protein was extracted from ileal mucosa samples using RIPA lysate (Servicebio Technology Co., Ltd., Wuhan, China) supplemented with 1% phenylmethanesulfonyl fluoride (PMSF). The separated proteins were transferred onto polyvinylidene fluoride (PVDF) membranes using SDS-PAGE; then, the membranes were blocked with a protein-free rapid blocking buffer (Epizyme Biomedicine Technology Co., Ltd., Shanghai, China) for 10 min. After washing 3–5 times, the membranes were incubated with diluted primary antibodies against β-actin, occluding, and ZO-1 (all from Servicebio Technology Co., Ltd., Wuhan, China) for 12 h at 4 °C. Following another 3–5 washes with PBST, the membranes were incubated with anti-rabbit IgG (Servicebio Technology Co., Ltd., Wuhan, China) for 1 h. Blots were scanned using a CanoScan LiDE 100 scanner (Canon, Tokyo, Japan), and quantification was performed with Image J software (version 1.8.0.112).

### 2.11. Assessment of Intestinal Histomorphology

Ileal and jejunal samples preserved in 4% buffered formaldehyde were sectioned and stained with hematoxylin and eosin; then, the crypt depth and villus height were observed and measured using a microscope (Eclipse 80i, Nikon Corporation, Tokyo, Japan) and the villus height to crypt depth ratio (V/C) was calculated [30].

### 2.12. Cultivation and Enumeration of Bacteria in Cecum

On a sterile operating table, cecal contents were finely fragmented, and 1 g was homogenized with 9 mL of phosphate-buffered saline. The mixture was then diluted to different gradient concentrations. A volume of 100 μL from each gradient concentration was plated onto the corresponding agar plates: total plate counts on plate count agar (PCA) (Solarbio Technology Co., Ltd., Qingdao, China), *Lactobacillus* on MRS agar (Solarbio Technology Co., Ltd., Qingdao, China), *Escherichia coli* on eosin TBX agar (Solarbio Technology Co., Ltd., Qingdao, China), and *Salmonella* on xylose lysine desoxycholate agar (XLD) (Solarbio Technology Co., Ltd., Qingdao, China). The MRS agar plates were plated under anaerobic conditions at 37 °C for 48 h, while the other agar plates were plated under aerobic conditions at 37 °C for 24 h. The microbial populations were quantified by plate counting and expressed as log_10_ CFU per gram of cecal content.

### 2.13. Data Collection and Statistical Analysis

Data management was performed using Excel (MS Excel 2021), and one-way analysis of variance (ANOVA) was conducted with SPSS Statistics 26.0 (SPSS Statistics, Chicago, IL, USA). The intestinal lesion scores and mortality were analyzed using the Kruskal–Wallis test. The remaining indices were analyzed using one-way ANOVA. Differences among treatments were evaluated using Duncan’s multiple range test, with results expressed as means. A *p*-value of less than 0.05 was considered statistically significant.

## 3. Results

### 3.1. The Chemical Constituents in CCHM

A total of 421 chemical constituents were identified in CCHM. Among these, berberine accounted for 33.11%, citric acid for 6.44%, 6-acetylcodeine for 5.58%, fraxetin for 1.44%, esculin for 1.33%, ethylmorphine for 1.31%, methoxyphenamine for 1.27%, D- (+)-pyroglutamic acid for 1.26%, D- (+)-malic acid for 1.19%, L-isoleucine for 0.98%, and other compound classes for 46.09% (Table 3, Figure S1).

### 3.2. Growth Performance

Broilers in the CL and CH groups had a lower ADG (*p* = 0.002; Table 4) and ADFI (*p* = 0.045) than those in the NC group, but were no differences compared to the AB and SE groups. The F:G ratio showed no differences among all groups from days 10 to 19 (*p* > 0.05). Broilers in the CL and CH groups had higher ADG and ADFI and a lower F:G ratio than those in the SE group from days 20 to 28 and days 0 to 28 (*p* < 0.05), but no differences were observed in the CL and CH groups compared to the AB and NC groups (*p* > 0.05). 

### 3.3. Intestinal Lesion Scores 

**Figure 1:**
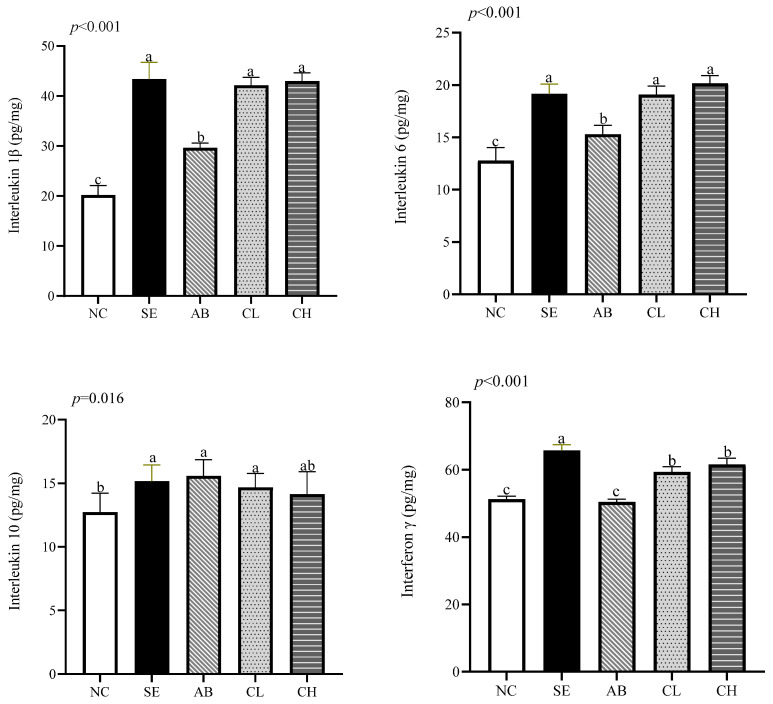

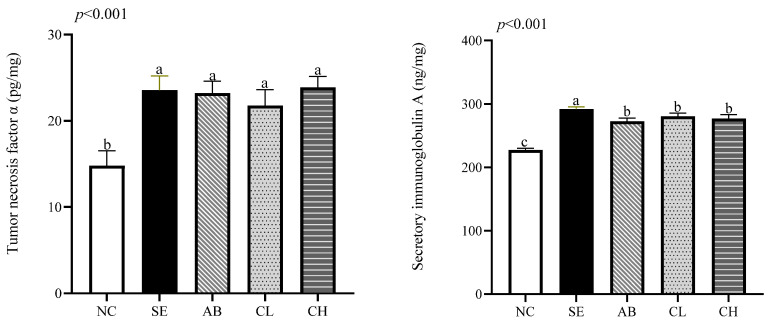
Effect of CCHM on the immune-related indices of the ileal mucosa of broilers infected with *Salmonella enteritidis* on day 19 (*n* = 6 per experimental group). ^a,b,c^ Different letters indicate differences (*p* < 0.05). NC, normal drinking water; SE, normal drinking water + *Salmonella enteritidis* challenge; AB, ciprofloxacin lactate injection (1 mL/L)-treated drinking water + *Salmonella enteritidis* challenge; CL, CCHM (5 mL/L)-treated drinking water + *Salmonella enteritidis* challenge; CH, CCHM (10 mL/L)-treated drinking water + *Salmonella enteritidis* challenge.

On days 19 and 28, the CL and CH groups had lower jejunal and ileal lesion scores compared to the SE group (*p* < 0.001, Figure 1, Table 5), but no differences were observed among the CL, CH, and AB groups (*p* > 0.05).

### 3.4. Immune Organ Indices

On days 19 and 28, the indices of the bursa of Fabricius and thymus in the CL and CH groups were higher than those in the SE group (*p* ≤ 0.001, Table 6), but no differences were observed among the CL, CH, and NC groups on day 28 (*p* > 0.05). The spleen index in the CL and CH groups was lower than that in the SE group on day 19 (*p* < 0.05), but no differences were observed among all groups on day 28 (*p* > 0.05).

### 3.5. Ileal Immune Function

**Figure 2:**
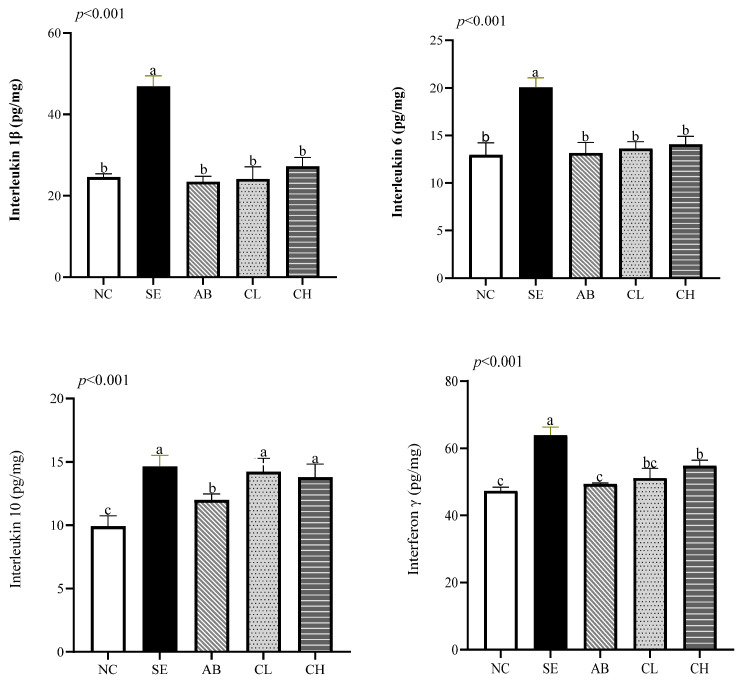

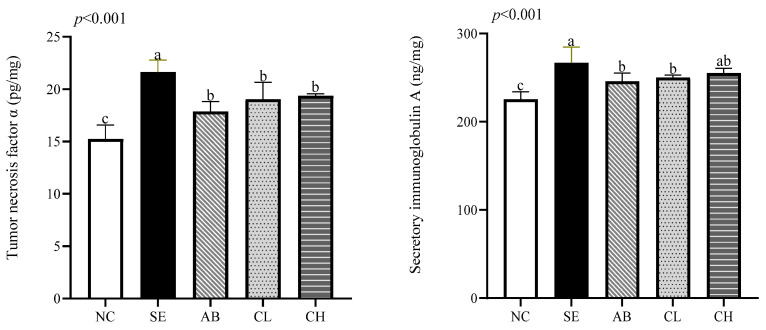
Effect of CCHM on the immune-related indices of the ileal mucosa of broilers infected with *Salmonella enteritidis* on day 28. (*n* = 6 per experimental group). ^a,b,c^ Different letters indicate differences (*p* < 0.05). NC, normal drinking water; SE, normal drinking water + *Salmonella enteritidis* challenge; AB, ciprofloxacin lactate injection (1 mL/L)-treated drinking water + *Salmonella enteritidis* challenge; CL, CCHM (5 mL/L)-treated drinking water + *Salmonella enteritidis* challenge; CH, CCHM (10 mL/L)-treated drinking water + *Salmonella enteritidis* challenge.

**Figure 3:**
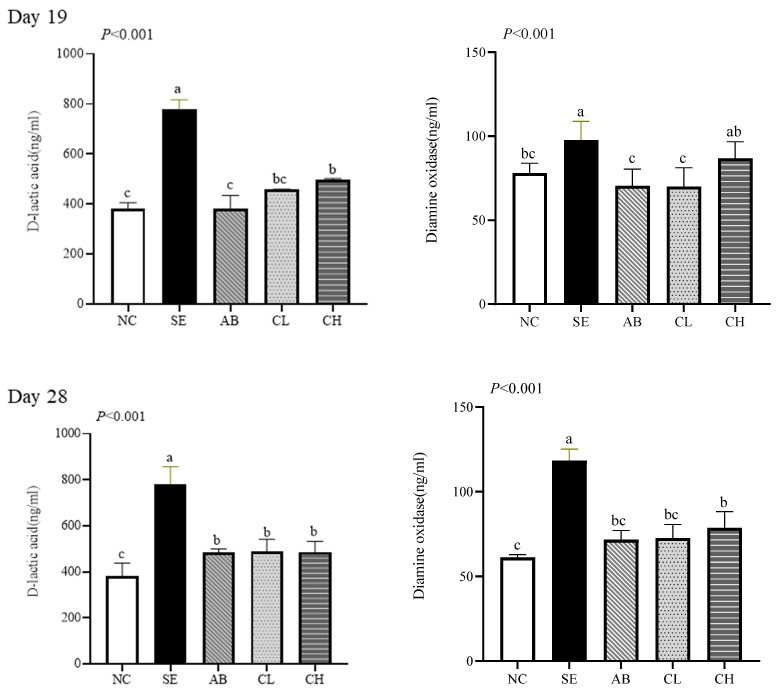
Effect of CCHM on *D*-lactic acid and diamine oxidase in broilers infected with *Salmonella enteritidis* (*n* = 6 per experimental group). ^a,b,c^ Different letters indicate differences (*p* < 0.05). NC, normal drinking water; SE, normal drinking water + *Salmonella enteritidis* challenge; AB, ciprofloxacin lactate injection (1 mL/L)-treated drinking water + *Salmonella enteritidis* challenge; CL, CCHM (5 mL/L)-treated drinking water + *Salmonella enteritidis* challenge; CH, CCHM (10 mL/L)-treated drinking water + *Salmonella enteritidis* challenge.

On day 19, the levels of TNF-α (*p* < 0.001, Figure 2), IFN-γ (*p* < 0.001), IL-1β (*p* < 0.001), IL-6 (*p* < 0.001), IL-10 (*p* = 0.016), and sIgA (*p* < 0.001) were higher in the SE group compared to the NC group, but there were no differences in the levels of TNF-α, IL-1β, IL-6, and IL-10 among the CL, CH, and SE groups (*p* > 0.05). On day 28, the levels of IL-1β (*p* < 0.001), IL-6 (*p* < 0.001), TNF-α (*p* < 0.001), and IFN-γ (*p* < 0.001) were lower in the CL and CH groups compared to the SE group (Figure 3). There were no differences in the levels of IL-1β, sIgA, TNF-α, and IL-6 among the CL, CH, and AB groups (*p* > 0.05), while the levels of IL-1β and IL-6 showed no differences among the CL, CH, AB, and NC groups on day 28 (*p* > 0.05).

### 3.6. Intestinal Permeability 

**Figure 4:**
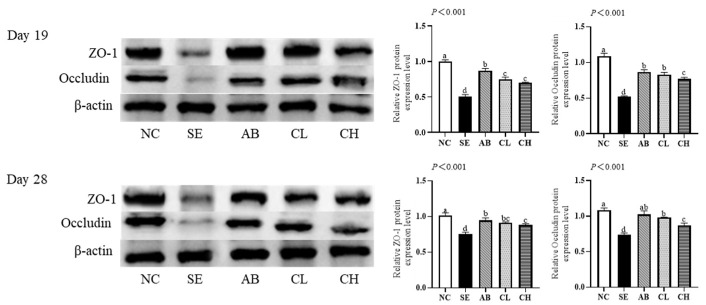
Effect of CCHM on the tight junction protein expressions of the ileal mucosa of broilers infected with *Salmonella enteritidis* (*n* = 6 per experimental group). ^a,b,c,d^ Different letters indicate differences (*p* < 0.05). NC, normal drinking water; SE, normal drinking water + *Salmonella enteritidis* challenge; AB, ciprofloxacin lactate injection (1 mL/L)-treated drinking water + *Salmonella enteritidis* challenge; CL, CCHM (5 mL/L)-treated drinking water + *Salmonella enteritidis* challenge; CH, CCHM (10 mL/L)-treated drinking water + *Salmonella enteritidis* challenge (Figure S2).

**Figure 5:**
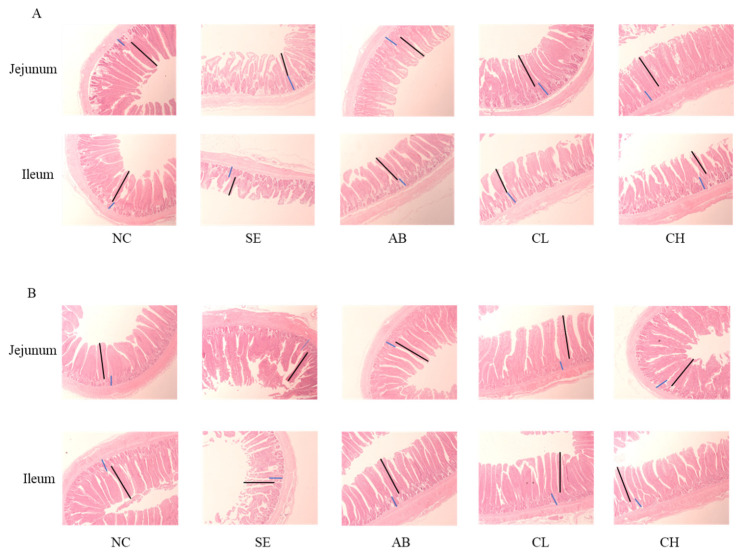
Effect of CCHM on intestinal morphology in broilers infected with *Salmonella enteritidis* (*n* = 6 per experimental group). (**A**,**B**) Jejunum and ileum tissues stained with hematoxylin and eosin on days 19 and 28 (scale bar: 200 μm). NC, normal drinking water; SE, normal drinking water + *Salmonella enteritidis* challenge; AB, ciprofloxacin lactate injection (1 mL/L)-treated drinking water + *Salmonella enteritidis* challenge; CL, CCHM (5 mL/L)-treated drinking water + *Salmonella enteritidis* challenge; CH, CCHM (10 mL/L)-treated drinking water + *Salmonella enteritidis* challenge. Villus height: black line; Crypt depth: blue line. Images of the transverse section were photographed under 50× magnification.

On days 19 and 28, the levels of serum *D*-lactic acid in the CL and CH groups were lower than in the SE group (*p* < 0.001, Figure 4). However, no differences were observed among NC, AB, and CL groups on day 19 (*p* > 0.05), and there were no differences among the CL, CH, and AB groups on day 28 (*p* > 0.05). The level of serum DAO (*p* < 0.001) in the CL group was lower than that in the SE group, but no differences were observed among the NC, AB, and CL groups on day 19 or 28 (*p* > 0.05). The ileal protein expressions of ZO-1 (*p* < 0.001) and occludin (*p* < 0.001) in the CL and CH groups were higher than those in the SE group, but no differences were observed among AB and CL groups on days 19 and 28 (*p* > 0.05, Figure 5).

### 3.7. Morphological Measurements of the Jejunum and Ileum

On day 19, the villus height and V/C value of the ileum and jejunum in the CL group were higher compared to those in the SE group and were lower compared to those in the NC group (*p* < 0.001, Figure 5 and Table 7), but there were no differences observed in the villus height of the jejunum and ileum and jejunal V/C value between the SE and CH groups (*p* > 0.05). The crypt depths of the ileum in the CL and CH groups were lower than those in the SE groups (*p* < 0.001), but no differences were found in the crypt depths of the jejunum between the CL, CH, and SE groups on day 19 (*p* > 0.05). On day 28, the villus height and V/C value of the ileum and jejunum in the CL and CH groups were higher compared to those in the SE group (*p* < 0.05), but there were no differences among the NC, AB, and CL groups (*p* > 0.05). The crypt depths of the jejunum and ileum in the CL and CH groups were lower compared to those in the SE group on day 28 (*p* < 0.001), but no differences were observed among the NC, AB, and CH groups (*p* > 0.05). 

### 3.8. Cecal Microbial Analysis

On days 19 and 28, the CL and CH groups exhibited lower counts of *Salmonella* and *Escherichia coli* compared to the SE group (*p* < 0.001, Table 8). However, no differences in *Salmonella* and *Escherichia coli* counts were observed among the NC, AB, CL, and CH groups on day 28 (*p* > 0.05). Additionally, the *Lactobacillus* count was lower in the SE group on days 19 and 28 (*p* < 0.001), with no differences between the AB and CL groups on day 28 (*p* > 0.05). The total plate count showed no differences between any groups on days 19 and 28 (*p* > 0.05).

## 4. Discussion

In the present study, the studied CCHM contained alkaloids (berberine), organic acids (citric acid), glycosides (esculin), and other active components. Berberine, citric acid, and esculin have been demonstrated to possess antimicrobial, anti-inflammatory, and growth-promoting properties [17,31,32]. Therefore, the improvements in growth performance, intestinal inflammation, and microbiota in broilers infected with *Salmonella enteritidis* and treated with CCHM may be related to its active constituents. 

In this study, broilers infected with *Salmonella enteritidis* showed higher mortality rates and increased intestinal lesion scores, along with a decrease in both ADG and ADFI. These findings suggested that intestinal injury caused by *Salmonella enteritidis* infection negatively impacted growth performance. However, the harmful effects were alleviated by CCHM, which was in line with the results reported by Stringfellow et al. [33], who found that citric acid reduced both intestinal lesions and mortality and improved growth performance upon the induction of necrotic enteritis in broilers via a *Clostridium perfringens* challenge. Previous studies showed that dietary supplementation with berberine and citric acid increased the ADG and ADFI to alleviate LPS-induced growth performance impairment in broilers [34,35].

The weight of immune organs serves as an indicator of the immune status of broilers, with higher immune organ indices correlating with enhanced immune function and reduced illness susceptibility [36]. In the present study, *Salmonella enteritidis* infection reduced the indices of the bursa of Fabricius, thymus, and spleen in broilers, which was consistent with findings reported by Sadeghi et al. [29]. However, supplementing CCHM into drinking water improved these indices, suggesting that CCHM promoted the growth of immune organs and enhanced the immunity of broilers. This effect may be attributed to the active constituents contained in CCHM, which promoted the development of the intestinal tract, thereby enhancing nutrient absorption and subsequently improving the organ index in broilers. A previous study showed that dietary supplementation with citric acid-rich *Schisandra chinensis* increased the ileal crude protein digestibility and indices of the spleen and thymus in broilers [37]. Furthermore, dietary supplementation with berberine in broilers increased the weights of the bursa of Fabricius, spleen, and thymus [38].

The morphology of the intestinal mucosa is a crucial indicator for assessing gut health, with shorter villi or deeper crypts being related to tissue damage caused by invasive pathogens [39]. *Salmonella enteritidis* infection could destroy the intestinal morphology of broilers, as evidenced by increased crypt depth and decreased villus height [40]. In this study, supplementing CCHM into drinking water improved the crypt depth, villus height, and V/C value of broilers infected with *Salmonella enteritidis*, indicating that CCHM alleviated the damage to intestinal villi induced by *Salmonella enteritidis*. According to a previous study, dietary supplementation with berberine decreased the crypt depth and increased the villus height and V/C ratio in broilers [41]. Similar results were observed with dietary supplementation of citric acid, which increased the intestinal villus height and V/C value in broilers [42].

Tight junction proteins are essential for maintaining intestinal barrier homeostasis, and their regulation is crucial for epithelial barrier repair after injury [43]. Compromise of the intestinal barrier causes the release of *D*-lactic acid and DAO into the bloodstream, making the measurement of their serum levels useful for assessing the degree of intestinal damage or recovery [44,45]. Our study showed that *Salmonella enteritidis* infection reduced ileal protein expressions of ZO-1 and occludin and increased levels of serum *D*-lactic acid and DAO. However, supplementing CCHM into drinking water alleviated these adverse effects, indicating that CCHM improved the intestinal barrier function. Yuan et al. [46] observed that berberine reduced the protein expressions of claudin-1 and occludin in the jejunum upon the induction of necrotic enteritis in broilers via a *Clostridium perfringens* challenge. Additionally, Bialkowski et al. [47] found that citric acid decreased the levels of serum *D*-lactic acid and DAO in broilers. 

Once a pathogen enters the colon and damages tissue, the intestinal epithelium releases pro-inflammatory mediators, which are closely associated with the intestinal inflammatory response [40]. The anti-inflammatory cytokine IL-10 reduces the transcription and secretion of pro-inflammatory cytokines in monocytes and macrophages [48]. In this study, *Salmonella enteritidis* infection led to increases in TNF-α, sIgA, IL-1β, IL-6, and IFN-γ levels in ileum. Supplementing CCHM into drinking water could reduce the release of these pro-inflammatory factors and increase the level of IL-10, indicating that CCHM could regulate immune factors to inhibit inflammation. Bialkowski et al. [47] discovered that citric acid supplementation reduced the mRNA expressions of TNF-α, IL-6, sIgA, and IFN-γ in broilers. Moreover, Shen et al. [49] reported that berberine reduced increases in pro-inflammatory markers such as IL-2, IL-12 and IFN-β induced by LPS injection in broilers.

The intestinal flora forms a multi-layered microbial barrier in the intestine, playing crucial roles in nutrition absorption, intestinal defense, and regulation of immune function [50,51]. In this study, *Salmonella enteritidis* infection increased the counts of *Salmonella* and *Escherichia coli* while decreasing the *Lactobacillus* count of broilers, indicating a disruption of the cecal microbiota. Supplementing CCHM into drinking water could increase the count of *Lactobacillus* and reduce the proliferation of harmful bacteria. Melaku et al. [52] found that dietary supplementation with citric acid promoted the proliferation of *Lactobacillus* in the caecum of broilers due to the decrease in intestinal pH. Several in vitro studies found that citric acid or esculin exhibited inhibitory effects against *Escherichia coli* and *Salmonella* [17,53]. Additionally, Yuan et al. [46] observed that berberine increased *Lactobacillus* counts and decreased the counts of Gram-negative bacteria such as *Clostridium perfringens* and *Escherichia coli* in broilers with *Clostridium perfringens* infection.

## 5. Conclusions

Supplementing CCHM into drinking water can improve the growth performance, intestinal barrier function, immune response, and cecal microflora of broilers infected with *Salmonella enteritidis*. In conclusion, CCHM can be used as a substitute for antibiotics to alleviate *Salmonella enteritidis* infection in broiler production. Under the presented experimental conditions, the optimal concentration of CCHM was 5 mL/L.

## Figures and Tables

**Table 1:** The timetable of the experimental design.

Groups	NC	SE	AB	CL	CH
Day of the start of the experiment	0	0	0	0	0
Number of broilers per group	60	60	60	60	60
Days of challenge with *Salmonella enteritidis*	-	9–11	9–11	9–11	9–11
Days of treatment with *ciprofloxacin*	-	-	12–18	-	-
Days of treatment with CCHM	-	-	-	12–18	12–18
Day of the end of the experiment	28	28	28	28	28
Number of evaluated broilers per group	6	6	6	6	6

**Table 2:** Ingredients and nutrient levels in the basal diet (air-dried basis) %.

Item	Contents	
	0–14 Days of Age	15–28 Days of Age
Ingredients		
Corn	54.72	60.25
Soybean meal	34.59	29.69
Fish meal	3.58	3.00
Soybean oil	3.73	3.80
Limestone	1.28	1.25
CaHPO_4_	0.43	0.30
NaCl	0.30	0.30
DL-Methionine	0.22	0.22
L-lysine	0.15	0.19
Premix ^1^	1.00	1.00
Total	100.00	100.00
Total nutrient levels ^2^		
Metabolizable energy (MJ/kg)	12.56	12.78
Crude protein, %	21.90	19.91
Calcium, %	0.92	0.82
Available phosphorus, %	0.61	0.46
Lysine, %	1.29	1.18
Methionine, %	0.58	0.55
Threonine, %	0.92	0.83

^1^ Provided per kilogram of diet: Cu (as copper sulfate) 15 mg; Mn (as manganese sulfate) 100 mg; Zn (as zinc sulfate) 100 mg; Se (as sodium selenite) 0.35 mg; Fe (as ferrous sulfate) 40 mg; I (as potassium iodide) 1.0 mg; vitamin A (trans-retinyl acetate) 10,000 IU; vitamin B_1_ (thiamin) 3.0 mg; vitamin B_2_ (riboflavin) 9.0 mg; vitamin B_6_ (pyridoxine HCl) 4.0 mg; vitamin B_12_ (cobalamin) 0.02 mg; vitamin D_3_ (cholecalciferol) 5000 IU; vitamin E (all-rac-α-tocopherol acetate) 80 IU; vitamin K_3_ (menadione) 3.0 mg; nicotinic acid 60 mg; calcium pantothenate 15 mg; folic acid 2.0 mg; biotin 0.15 mg. ^2^ The nutrient levels were calculated values.

**Table 3:** The chemical constituents in CCHM.

No	Compounds	Formula	Content (%)
1	Berberine	C_20_H_17_NO_4_	33.11
2	Citric acid	C_6_H_8_O_7_	6.44
3	6-Acetylcodeine	C_20_H_23_NO_4_	5.58
4	Fraxetin	C_10_H_8_O_5_	1.44
5	Esculin	C_15_H_16_O_9_	1.33
6	Ethylmorphine	C_19_H_23_NO_3_	1.31
7	Methoxyphenamine	C_11_H_17_NO	1.27
8	D- (+)-Pyroglutamic acid	C_5_H_7_NO_3_	1.26
9	D- (+)-Malic acid	C_4_H_6_O_5_	1.19
10	L-Isoleucine	C_6_H_13_NO_2_	0.98
11	Others		46.09

**Table 4:** Effect of CCHM on growth performance in broilers infected with *Salmonella enteritidis* from days 0 to 28 (*n* = 6 per experimental group).

Items ^1^	NC	SE	AB	CL	CH	*p*-Value
Day 0–9						
Average daily gain (g)	10.31 ± 0.48	10.53 ± 0.23	10.32 ± 0.22	10.59 ± 0.36	10.75 ± 0.19	0.918
Average daily feed intake (g)	16.32 ± 1.23	16.41 ± 1.87	15.78 ± 1.39	15.83 ± 1.96	15.81 ± 2.34	0.854
Feed/gain ratio	1.58 ± 0.03	1.53 ± 0.03	1.52 ± 0.03	1.49 ± 0.02	1.51 ± 0.03	0.727
Day 10–19						
Average daily gain (g)	42.59 ± 0.97 ^a^	37.51 ± 1.03 ^b^	38.97 ± 0.91 ^b^	37.67 ± 0.36 ^b^	37.85 ± 1.20 ^b^	0.002
Average daily feed intake (g)	70.70 ± 2.13 ^a^	66.62 ± 1.71 ^b^	65.88 ± 0.36 ^b^	66.50 ± 0.72 ^b^	66.26 ± 0.56 ^b^	0.045
Feed/gain ratio	1.66 ± 0.02	1.78 ± 0.03	1.70 ± 0.04	1.77 ± 0.02	1.76 ± 0.05	0.077
Day 20–28						
Average daily gain (g)	50.02 ± 1.45 ^a^	42.54 ± 1.25 ^b^	50.58 ± 1.64 ^a^	51.16 ± 1.40 ^a^	49.84 ± 2.15 ^a^	<0.001
Average daily feed intake (g)	87.03 ± 0.67 ^a^	78.27 ± 0.38 ^b^	88.52 ± 1.63 ^a^	88.51 ± 1.08 ^a^	88.22 ± 0.76 ^a^	<0.001
Feed/gain ratio	1.74 ± 0.01 ^b^	1.84 ± 0.01 ^a^	1.75 ± 0.01 ^b^	1.73 ± 0.02 ^b^	1.77 ± 0.02 ^b^	0.001
Day 0–28						
Average daily gain (g)	34.60 ± 0.22 ^a^	30.38 ± 0.27 ^c^	33.34 ± 0.12 ^b^	33.02 ± 0.27 ^b^	32.90 ± 0.42 ^b^	<0.001
Average daily feed intake (g)	58.13 ± 0.7 1^a^	53.78 ± 0.0.37 ^b^	56.67 ± 0.36 ^a^	56.80 ± 0.52 ^a^	56.92 ± 0.24 ^a^	<0.001
Feed/gain ratio	1.68 ± 0.01 ^b^	1.77 ± 0.01 ^a^	1.70 ± 0.02 ^b^	1.72 ± 0.01 ^b^	1.73 ± 0.02 ^ab^	0.006
Mortality (%)	0.00 ± 0.00 ^b^	5.50 ± 0.22 ^a^	0.17 ± 0.17 ^b^	0.33 ± 0.21 ^b^	0.50 ± 0.22 ^b^	0.043

^a,b,c^ Different letters indicate differences (*p* < 0.05). ^1^ NC, normal drinking water; SE, normal drinking water + *Salmonella enteritidis* challenge; AB, ciprofloxacin lactate injection (1 mL/L)-treated drinking water + *Salmonella enteritidis* challenge; CL, CCHM (5 mL/L)-treated drinking water + *Salmonella enteritidis* challenge; CH, CCHM (10 mL/L)-treated drinking water + *Salmonella enteritidis* challenge.

**Table 5:** Effect of CCHM on intestinal lesion scores in broilers infected with *Salmonella enteritidis* (*n* = 6 per experimental group).

Items ^1^	NC	SE	AB	CL	CH	*p*-Value
Day 19						
Jejunum lesion score ^2^	0.00 ± 0.00 ^c^	4.83 ± 0.31 ^a^	1.00 ± 0.37 ^b^	1.16 ± 0.31 ^b^	1.67 ± 0.21 ^b^	<0.001
Ileum lesion score ^2^	0.00 ± 0.00 ^c^	4.67 ± 0.21 ^a^	0.83 ± 0.31 ^b^	1.16 ± 0.31 ^b^	1.50 ± 0.22 ^b^	<0.001
Day 28						
Jejunum lesion score ^2^	0.00 ± 0.00 ^c^	5.17 ± 0.31 ^a^	0.50 ± 0.22 ^bc^	0.83 ± 0.31 ^b^	1.00 ± 0.26 ^b^	<0.001
Ileum lesion score ^2^	0.00 ± 0.00 ^c^	4.67 ± 0.21 ^a^	0.50 ± 0.22 ^bc^	0.67 ± 0.21 ^b^	0.67 ± 0.21 ^b^	<0.001

^a,b,c^ Different letters indicate differences (*p* < 0.05). ^1^ NC, normal drinking water; SE, normal drinking water + *Salmonella enteritidis* challenge; AB, ciprofloxacin lactate injection (1 mL/L)-treated drinking water + *Salmonella enteritidis* challenge; CL, CCHM (5 mL/L)-treated drinking water + *Salmonella enteritidis* challenge; CH, CCHM (10 mL/L)-treated drinking water + *Salmonella enteritidis* challenge. ^2^ The higher the score, the more villus damage.

**Table 6:** Effect of CCHM on the values for immune organ indices in broilers infected with *Salmonella enteritidis* (*n* = 6 per experimental group).

Items ^1^	NC	SE	AB	CL	CH	*p*-Value
Day 19						
Thymus index (g/kg)	1.73 ± 0.05 ^a^	1.28 ± 0.08 ^c^	1.75 ± 0.06 ^a^	1.67 ± 0.05 ^ab^	1.55 ± 0.04 ^b^	<0.001
Spleen index (g/kg)	2.90 ± 0.05 ^a^	2.40 ± 0.09 ^c^	2.94 ± 0.07 ^a^	2.83 ± 0.06 ^a^	2.59 ± 0.02 ^b^	<0.001
Bursa of Fabricius index (g/kg)	1.36 ± 0.03 ^ab^	1.07 ± 0.06 ^c^	1.48 ± 0.05 ^a^	1.37 ± 0.03 ^a^	1.23 ± 0.06 ^b^	<0.001
Day 28						
Thymus index (g/kg)	1.36 ± 0.02 ^ab^	1.05 ± 0.05 ^c^	1.42 ± 0.06 ^a^	1.36 ± 0.03 ^ab^	1.29 ± 0.03 ^b^	<0.001
Spleen index (g/kg)	4.01 ± 0.08	3.54 ± 0.07	4.05 ± 0.10	3.90 ± 0.09	3.93 ± 0.17	0.068
Bursa of Fabricius index (g/kg)	1.15 ± 0.05 ^a^	0.77 ± 0.08 ^b^	1.16 ± 0.05 ^a^	1.16 ± 0.10 ^a^	1.03 ± 0.05 ^a^	0.001

^a,b,c^ Different letters indicate differences (*p* < 0.05). ^1^ NC, normal drinking water; SE, normal drinking water + *Salmonella enteritidis* challenge; AB, ciprofloxacin lactate injection (1 mL/L)-treated drinking water + *Salmonella enteritidis* challenge; CL, CCHM (5 mL/L)-treated drinking water + *Salmonella enteritidis* challenge; CH, CCHM (10 mL/L)-treated drinking water + *Salmonella enteritidis* challenge.

**Table 7:** Effect of CCHM on intestinal morphology in broilers infected with *Salmonella enteritidis* (*n* = 6 per experimental group).

Items ^1^	NC	SE	AB	CL	CH	*p*-Value
Day 19						
Jejunum						
Villus height (µm)	1110.51 ± 17.36 ^a^	837.58 ± 33.49 ^c^	1043.6 ± 31.20 ^ab^	976.29 ± 43.85^b^	868.44 ± 31.29 ^c^	<0.001
Crypt depth (µm)	167.49 ± 9.86 ^c^	264.80 ± 11.26 ^a^	199.87 ± 2.37 ^b^	244.50 ± 11.01^a^	253.71 ± 7.02 ^a^	<0.001
Villus height/crypt depth value	6.70 ± 0.29 ^a^	3.16 ± 0.03 ^d^	5.22 ± 0.17 ^b^	4.01 ± 0.18^c^	3.44 ± 0.22 ^cd^	<0.001
Ileum						
Villus height (µm)	909.25 ± 14.53 ^a^	727.22 ± 14.14 ^b^	875.30 ± 19.62 ^a^	763.25 ± 21.62 ^b^	754.87 ± 25.36 ^b^	<0.001
Crypt depth (µm)	150.40 ± 3.57 ^c^	235.99 ± 13.18 ^a^	182.55 ± 6.85 ^b^	205.80 ± 9.41 ^b^	208.94 ± 9.35 ^b^	<0.001
Villus height/crypt depth value	6.06 ± 0.16 ^a^	3.13 ± 0.21 ^d^	4.81 ± 0.08 ^b^	3.72 ± 0.09 ^c^	3.62 ± 0.09 ^c^	<0.001
Day 28						
Jejunum						
Villus height (µm)	1266.70 ± 46.70 ^a^	914.54 ± 38.28 ^c^	1182.03 ± 63.13 ^ab^	1232.96 ± 44.87 ^ab^	1120.78 ± 17.17 ^b^	<0.001
Crypt depth (µm)	199.08 ± 8.49 ^c^	297.28 ± 9.43 ^a^	210.71 ± 3.56 ^c^	199.38 ± 8.53 ^c^	234.10 ± 4.45 ^b^	<0.001
Villus height/crypt depth value	6.44 ± 0.48 ^a^	3.08 ± 0.10 ^c^	5.64 ± 0.32 ^ab^	6.22 ± 0.31 ^a^	4.79 ± 0.06 ^b^	<0.001
Ileum						
Villus height (µm)	1003.22 ± 49.47 ^a^	784.07 ± 20.65 ^b^	1010.25 ± 33.77 ^a^	993.18 ± 47.92 ^a^	934.44 ± 16.17 ^a^	0.001
Crypt depth (µm)	196.19 ± 5.56 ^b^	246.11 ± 3.90 ^a^	196.36 ± 2.63 ^b^	200.17 ± 3.83 ^b^	210.66 ± 8.61 ^b^	<0.001
Villus height/crypt depth value	5.11 ± 0.18 ^a^	3.19 ± 0.08 ^c^	5.15 ± 0.16 ^a^	4.95 ± 0.16 ^a^	4.45 ± 0.11 ^b^	<0.001

^a,b,c,d^ Different letters indicate differences (*p* < 0.05). ^1^ NC, normal drinking water; SE, normal drinking water + *Salmonella enteritidis* challenge; AB, ciprofloxacin lactate injection (1 mL/L)-treated drinking water + *Salmonella enteritidis* challenge; CL, CCHM (5 mL/L)-treated drinking water + *Salmonella enteritidis* challenge; CH, CCHM (10 mL/L)-treated drinking water + *Salmonella enteritidis* challenge.

**Table 8:** Effect of CCHM on bacterial counts in the cecum of broilers infected with *Salmonella enteritidis* (*n* = 6 per experimental group).

Items ^1^	NC	SE	AB	CL	CH	*p*-Value
Day 19						
Total plate count (log_10_ CFU/g)	10.83 ± 0.05	10.85 ± 0.04	10.79 ± 0.04	10.83 ± 0.06	10.85 ± 0.03	0.952
*Lactobacillus* (log_10_ CFU/g)	9.90 ± 0.01 ^a^	9.35 ± 0.02 ^d^	9.51 ± 0.02 ^c^	9.61 ± 0.02 ^b^	9.59 ± 0.01 ^b^	<0.001
*Salmonella* (log_10_ CFU/g)	4.77 ± 0.01 ^b^	5.97 ± 0.01 ^a^	4.49 ± 0.02 ^c^	4.53 ± 0.02 ^c^	4.51 ± 0.01 ^c^	<0.001
*Escherichia coli* (log_10_ CFU/g)	4.10 ± 0.06 ^c^	5.95 ± 0.01 ^a^	4.61 ± 0.03 ^b^	4.60 ± 0.02 ^b^	4.61 ± 0.01 ^b^	<0.001
Day 28						
Total plate count (log_10_ CFU/g)	10.99 ± 0.06	11.04 ± 0.04	11.02 ± 0.03	10.97 ± 0.06	10.99 ± 0.04	0.590
*Lactobacillus* (log_10_ CFU/g)	10.34 ± 0.02 ^a^	9.81 ± 0.01 ^d^	10.13 ± 0.05 ^b^	10.07 ± 0.02 ^b^	9.97 ± 0.01 ^c^	<0.001
*Salmonella* (log_10_ CFU/g)	5.44 ± 0.01 ^b^	7.85 ± 0.21 ^a^	5.46 ± 0.01 ^b^	5.46 ± 0.04 ^b^	5.55 ± 0.01 ^b^	<0.001
*Escherichia coli* (log_10_ CFU/g)	4.36 ± 0.02 ^b^	6.11 ± 0.04 ^a^	4.45 ± 0.02 ^b^	4.42 ± 0.04 ^b^	4.44 ± 0.03 ^b^	<0.001

^a,b,c,d^ Different letters indicate differences (*p* < 0.05). ^1^ NC, normal drinking water; SE, normal drinking water + *Salmonella enteritidis* challenge; AB, ciprofloxacin lactate injection (1 mL/L)-treated drinking water + *Salmonella enteritidis* challenge; CL, CCHM (5 mL/L)-treated drinking water + *Salmonella enteritidis* challenge; CH, CCHM (10 mL/L)-treated drinking water + *Salmonella enteritidis* challenge.

## Data Availability

All data sets collected and analyzed in this study are available at the request of the corresponding authors.

## Supplementary Materials

The following supporting information can be downloaded at: https://www.mdpi.com/article/10.3390/ani14182670/s1, Figure S1: Total ion current spectrum of CCHM natural product identification; Figure S2: Western blots raw data.
