# Supplementary material for: Effects of a Combined Chinese Herbal Medicine on Growth Performance, Intestinal Barrier Function, Immune Response, and Cecal Microflora in Broilers Infected with Salmonella enteritidis
Source: Animals (Basel). 2024 Sep 13;14(18):2670. doi: 10.3390/ani14182670 (PMC11429040; doi:10.3390/ani14182670)

Supplementary Figure S1. Total ion current spectrum of CCHM natural product identification

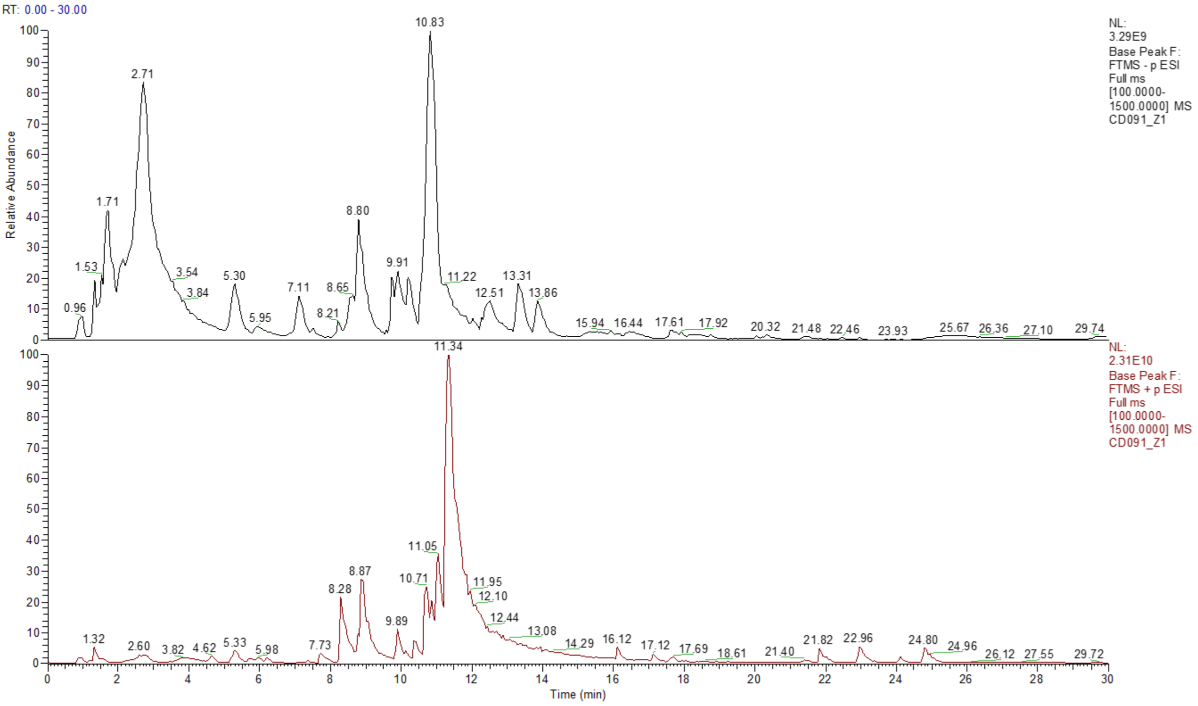

Black is the total ion flow diagram of negative ion mode; red is the total ion flow diagram of positive ion mode.

Supplementary Figure S2. Western blots raw data

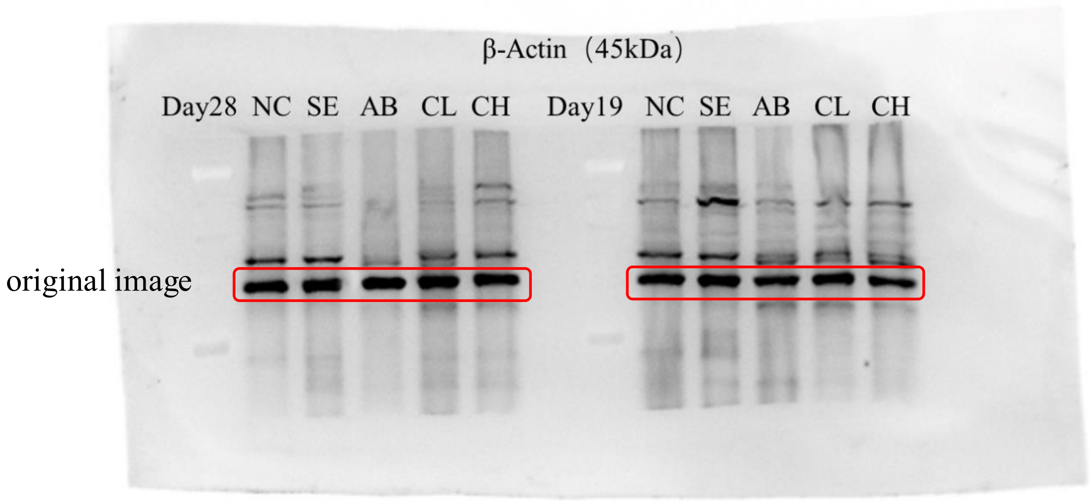

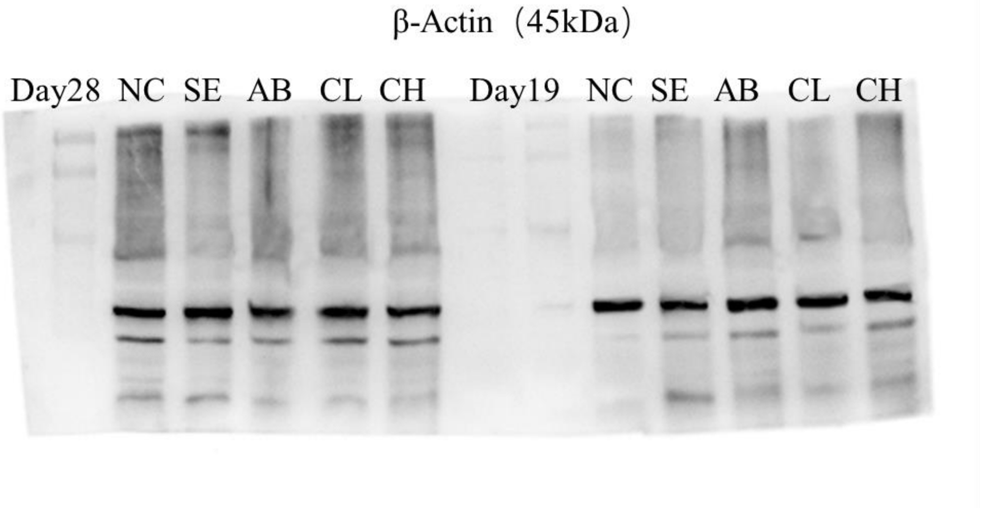

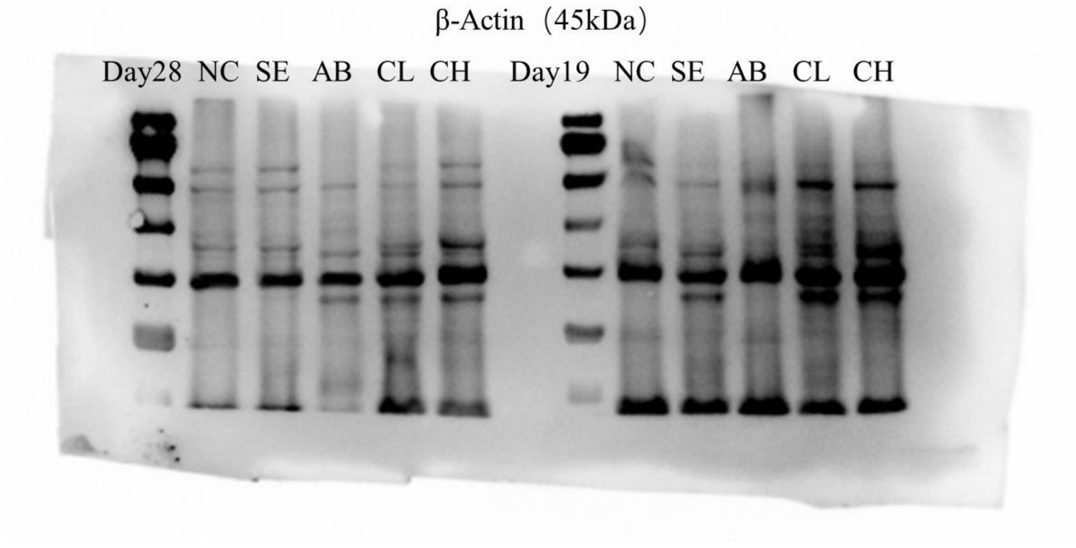

Occlucdin（59kDa）

Day19 NC SE AB CL CH Day28 NC SE AB CL CH

original image

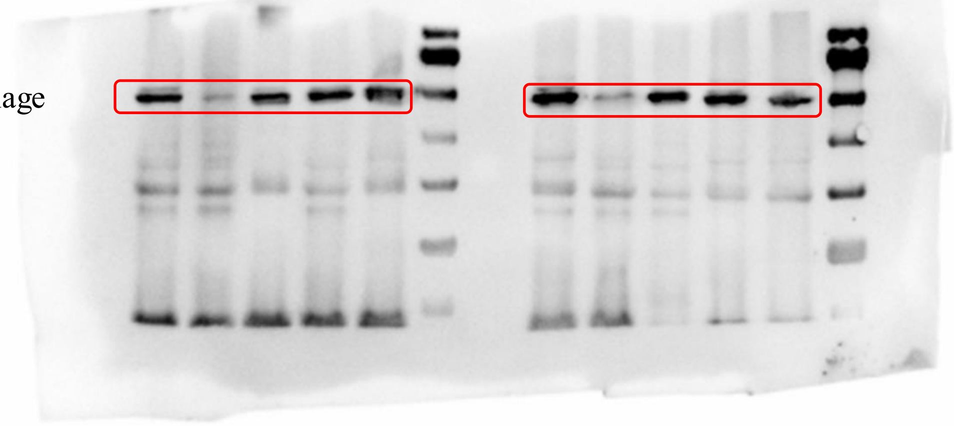

Occlucdin（59kDa）

Day19 NC SE AB CL CH Day28 NC SE AB CL CH

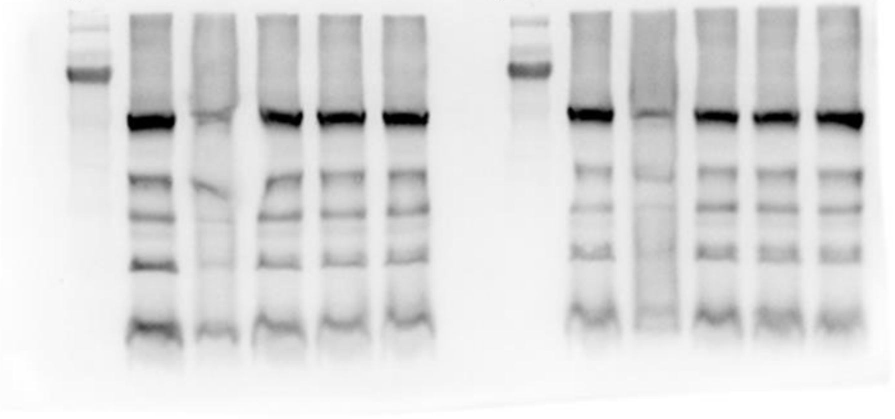

Occlucdin（59kDa）

Day19 NC SE AB CL CH Day28 NC SE AB CL CH

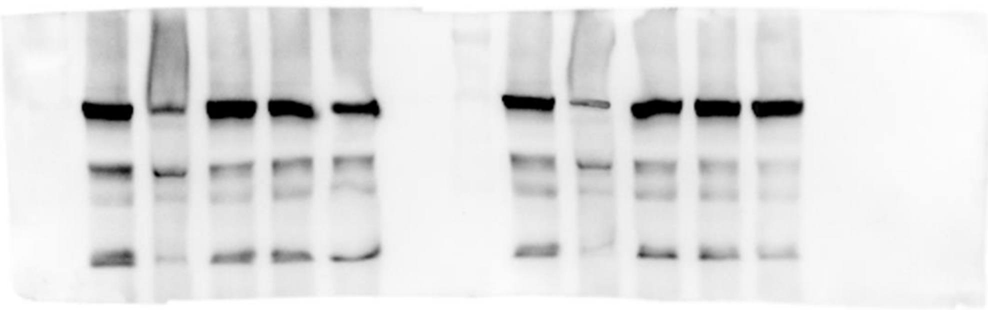

ZO-1（195kDa）

original image

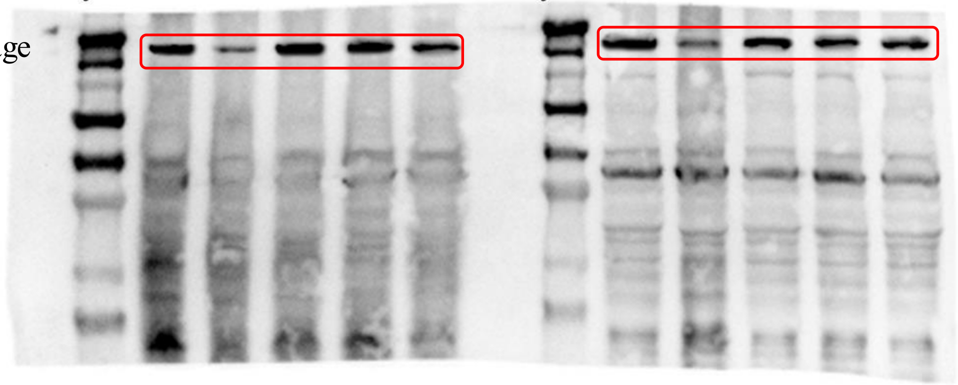

ZO-1（195kDa）

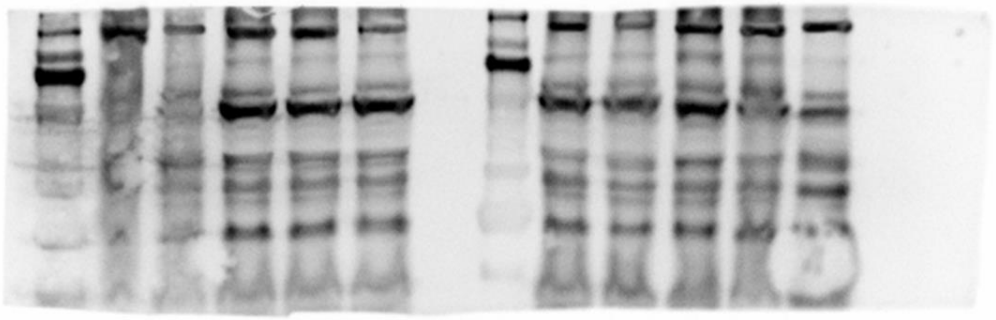

ZO-1（195kDa）

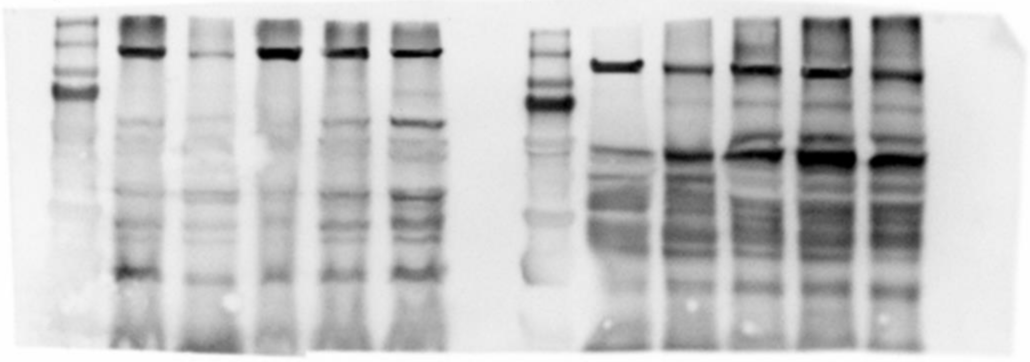

Supplement: Supplementary file 1 [file animals-14-02670-s001.zip › animals-3174758-supplementary.pdf]
